# Do Ocular Fluids Represent a Transmission Route of SARS-CoV-2 Infection?

**DOI:** 10.3389/fmed.2020.620412

**Published:** 2021-01-05

**Authors:** Giulio Petronio Petronio, Roberto Di Marco, Ciro Costagliola

**Affiliations:** Department of Medicine and Health Science “V. Tiberio”, Università degli Studi del Molise, Campobasso, Italy

**Keywords:** COVID-19, ocular fluids, transmission route, SARS-CoV-2, healthcare protection

## Abstract

The spread of the new SARS-CoV-2 is marked by a short timeline. In this scenario, explaining or excluding the possible transmission routes is mandatory to contain and manage the spread of the disease in the community. In the recent pandemic, it is still unclear how coronavirus can end up in ocular fluids. Nevertheless, eye redness and irritation in COVID-19 patients have been reported, suggesting that a possible ocular manifestation of SARS-CoV-2 infection may be conjunctivitis. On the basis of epidemiological data provided by previous SARS-Cove infection, numerous theories have been proposed: (1) conjunctiva as the site of direct inoculation by infected droplets; (2) the nasolacrimal duct as a migration route of the virus to the upper respiratory tract, or (3) haematogenic infection of the tear gland. The demand for further investigations to verify ocular involvement in COVID-19 infection came out from the results of recent meta-analysis studies, so the eye cannot be completely excluded as a transmission route of the infection. Thus, healthcare personnel and all the people that enter in contact with infected or suspected patients must always use the prescribed protective equipment.

## Introduction

The first cases of Severe Acute Respiratory Syndrome Coronavirus 2 (SARS-CoV-2) were directly linked to an animal market in Wuhan, China. To date, the virus disease clinical evidence such as symptoms and pathogenesis, as well as the systemic inflammatory response have been widely investigated by the scientific community (1).

Despite this, studies of the specific viral pathogenetic mechanisms in different human tissues are still a very broad subject that demands further considerations. In this regard, scientific evidence about the role of conjunctiva and ocular fluids as possible routes of transmission is still few and not conclusive.

For these reasons, the aim of this narrative review was to explore the role of conjunctiva and ocular fluids in the transmission of SARS-CoV-2 infection by a literature research focused on the most recent and relevant scientific publications.

### The Epidemiological Data: The Italian Experience

On 30th Jan 2020, the WHO declared the coronavirus epidemic, and on 28th Feb, it raised the threat to the coronavirus epidemic to a “very high” level worldwide. On 11th Mar, WHO Director-General, on evidence that the SARS-CoV-2 was not confined to some geographical regions, stated the pandemic spread throughout the planet. On 13th Mar, Europe was becoming the new epicenter of the pandemic. The first cases of SARS-CoV-2 reported in Europe date back to 30th Jan at the Spallanzani Institute (Rome, Italy). The first case of secondary transmission also occurred in Italy, in Codogno, Municipality of Lombardy in the province of Lodi, on 18th Feb. In a period of more than 3 months (from 13th May to 29th November), Italy ranked from 3th to 7th for the number of COVID-19 cases worldwide (2) with 1,585,178 cases reported, with 54,904 deceased, 734,503 dismissed/healed and 795,771 positives. According to the Italian Ministry of Health, COVID-19 percentage case fatality rate (CFR) by age group are: age group 0–9, 0% CFR; age group 10–19, 0% CFR; age group 20–29, 0.01% CFR; age group 30–39, 0.06% CFR; age group 40–49, 0.19% CFR; age group 50–59, 0.64% CFR; age group 60–69, 3% CFR; age group 70–79, 28% CFR; age group 80–89 18.86% CFR; age group ≥90 22.37% CFR (data available for 1,545,752 cases). The highest CFR % value was found in the over-90s age group (22.37%), this figure is significantly higher than the median age of cases (48 years) and the presence of comorbidity (3).

Based on available data on November 25th 2020 from Italian Ministry of Health, the most frequently observed symptoms before hospitalization in deceased patients are: fever 71%, dyspnoea 73%, cough 34%, diarrhea 6%, and hemoptysis 1%. Patients with 3 or more pre-existing pathologies are 65.7%, followed by 2 pre-existing pathologies (18.5%) and 1 pre-existing pathology (12.6%). The percentage of patients without pre-existing pathologies is very low (3.2%), demonstrating that the presence of comorbidities strongly influences the infection *exitus* (4).

The deaths in Milan, during the period January/March was of 3,888 people in 2019, whereas it has reached the number of 4,459 people during the same period of 2020, with a rise of 14.7%. Data becomes even more appalling if one considers the period March 1st−31st, with an increase of 76,1% compared to the same period of 2019. The higher mortality rate recorded in Italy over other countries can be explained, in part, by the older age distribution of the infected patients (5). These epidemiological data, together with those coming from other EU countries and United Kingdom, have allowed the formulation of the Risk Assessment by the European Center for Disease Prevention and Control (ECDC), that pointed out how the risk of severe disease associated with SARS-CoV-2 infection for people in Europe is mild for the population and high for the seniors and those with chronic illnesses (6).

### Transmission Route

Looking at what is happening worldwide, it has been realized that the spread of the new SARS-CoV-2 is marked by a short timeline. In this scenario, explaining or excluding the possible transmission routes is mandatory to contain and manage the spread of the disease in the community. Until now, not all the transmission routes of SARS-CoV-2 are known. Nevertheless, the primary mode of infection for SARS CoV-2 is the human-to-human transmission by droplets and direct contacts, as previously recognized for CoVs infections, such as SARS Cove and MERS Cove (7). Beyond this modality, recent studies conducted on specimens from multiple sites of 205 patients with SARS-CoV-2, detected the live virus in feces, implying that SARS-CoV-2 may also be transmitted through the fecal route (8). Furthermore, a possible systemic involvement has been suggested, given the presence of a small percentage of blood samples with positive results (9). In one case, the passage of the virus into the peritoneal cavity and fluids was also reported. This discovery raised concerns about the risks of exposure and contagion for the entire surgical staff, since all patients potentially, even those with mild respiratory symptoms, could present a viral load in the peritoneal fluid (10). A further hypothesis involves vectors such as domestic animals, flies, mosquitoes, or *Demodex folliculorum* in skin-to-skin transmission, as well as the direct human-to-human transmission (11). Thus, it would be helpful to cut nails as short as possible, to cut hair or to keep them tied back, and to shave beards, taking into account that sebum secretion too can be contaminated by the virus. In this context, disinfection of all instruments used for personal hygiene before and after use is strictly necessary and should not be shared to limit the spread of the virus (11).

#### Ocular Fluids

To date, it is still unclear how coronavirus can end up in ocular fluids, although feline and murine models have been used to record clinical manifestations such as conjunctivitis, anterior uveitis, retinitis, and optic neuritis (12). In the recent pandemic, a hypothesis about humans conjunctivitis as a possible ocular manifestation of SARS-CoV-2 infection was also made. This finding is partly supported by numerous clinical evidence, such as redness and eye irritation in patients with COVID-19. On the basis on epidemiological data provided by previous SARS-CoV infection, numerous theories have been proposed: (1) conjunctiva as the site of direct inoculation by infected droplets; (2) the nasolacrimal duct as a migration route of the virus to the upper respiratory tract; or (3) haematogenic infection of the tear gland (13).

New evidence about the novel coronavirus SARS-CoV-2 affecting the human eye has been reported. The main receptor of COVID-19 host cells that plays a crucial role in the entry of the virus into the cell to cause the final infection is the angiotensin 2 conversion enzyme (ACE2) (14). The expression of ACE2 in the conjunctiva (together with the epithelial cells of the lung, intestines, kidney, blood vessels), could indicate a potential infection route of the virus via these tissues (15). The presence of ACE2 receptors in human ocular tissue and CD147 in ocular fluids strongly suggests a role toward SARS-CoV-2 at the ocular level, consisting in the facilitation of virus entry inside the cell, followed by its replication and release (16).

Despite the presence of these facilitating mechanisms, ocular manifestations of COVID-19 are overall rare in the published literature. According to a study published on 30th Apr 2020, by Guan et al. (17) <1% of patients across 30 provinces in China (out of 33 of the total) were reported to have a conjunctiva involvement.

The demand for further investigations to verify ocular involvement in COVID-19 infection came out from the results of five reviews conducted by Sarma et al. (18), Loffredo et al. (19), Siedlecki et al. (20), Emparan et al. (21), and Torres-Costa (22) that aimed to demonstrate a correlation between the manifestation of ocular symptoms and the occurrence of systemic ones.

On 20th Mar 2020, Sarma et al. screened 5 different literature databases (PubMed, Google Scholar, EMBASE, Medrixv, and BioRixv). In their systematic review and meta-Analysis, authors included studies about the ocular manifestation of SARS-COV-2 patients were without language restriction. It is interesting to highlight how the authors applied two different eligibility criteria, depending on the type of study conducted. Indeed, for systematic review, different types of study (i.e., case report, case series) were included along with observations and any other type of study design that reported an ocular manifestation or its possible complication due to viral infection. On the other hand, for the meta-analysis study, only observational studies that included patients with Novel Coronavirus Pneumonia (confirmed by clinical or laboratory tests or both) were reported.

Loffredo et al. evaluated the frequency of conjunctivitis in patients affected by severe and non-severe COVID-19 infection according to the PRISMA (Preferred Reporting Items for Systematic Reviews and Meta-Analysis) only on clinical studies identified by searching Pubmed, ISI Web of Science, SCOPUS, and Cochrane electronic databases. On 5th Apr 2020, authors included 1,167 COVID-19 patients in their meta-analysis (19).

Siedlecki et al. used the PubMed.gov for searching relevant articles. On 16th Apr 2020, authors identified more than 20 articles on the ophthalmological aspects of COVID-19, of these, close to 60% were from Asia, around 30% were from the USA, and <15% were from Europe. The authors have analyzed different types of scientific articles, including original studies, letters, case studies, and reviews (20).

On 29th May 2020, Emparan et al. published a structured review on COVID-19 and ophthalmology using PubMed, ScienceDirect, LILACS, SciELO, the Cochrane Library, and Google Scholar as electronic databases. The Oxford Center for Evidence-Based Medicine 2011 Levels of Evidence worksheet was employed by authors for quality assessments. More than 1,000 manuscripts were identified in the research; only 26 records were included in the qualitative synthesis and of these only 17 were classified as level 5 within the classification system of the Oxford CBME methodology, the rest were level 4 (21).

Lastly, on 16th Jun 2020, Torres-Costa et al. reviewed the most relevant articles together with the official recommendations of ophthalmological societies by literature search on PubMed electronic database.

Despite the different research strategies and bibliographic analysis methods used by the authors, all the studies concluded that keratoconjunctivitis is the most representative ocular finding and that there is a correlation between the severity of the infection and eye involvement. The eye can be both an active (through tears) and passive (via the nasolacrimal duct) infection pathway. Tear film represents a natural defensive barrier against pathogens, thanks to the presence of antimicrobial proteins (such as lactoferrin, lysozyme, lipocalin, and beta-lysine) and immunoglobulins (23). Specifically, lactoferrin inhibits the virus binding protein by preventing the attachment of SARS-CoV to heparan sulfate proteoglycans (24). In addition, the possibility of foreign particles adhering and potentially invading epithelial cells is significantly reduced by the continuous rinsing of tears from the anterior surface of the eye to the nasolacrimal system together with the thick layer of mucin above the epithelium (25). Lastly, the outer lipid layer of tear film also enhances the resistance to the pathogen invasion. The lack of this layer in the mucosal membrane of the nasal and respiratory tract might explain the high affinity of Covid-19 for respiratory tract compared to that observed for the anterior ocular surface. Contrarily, a degraded anterior ocular surface, like in dry eye, could facilitate the viral infection into ocular and nasopharyngeal tissues (26). Indeed, dry eye is characterized by an abnormal tear film, through a broad spectrum of anomalies which varies from tear deficiency to atypical tear composition. The final result consists of a disruption of the tear film homeostasis that adversely affects the ability both to perform essential physiologic functions and to prevent microbial invasion (27). Especially the anatomical and physiological properties of the eyes may explain the discrepancy between the theoretically expected high rate of eye surface inflammation due to viral infection and the relatively low clinical incidence observed. Among these, the role played by the electrical standing potential of the eye in repelling aerosol particles (microdroplets) from the ocular surface should also be considered in terms of prevention of ocular infections (28).

On 17th Apr 2020, Colavita et al. (29) observed an early ocular involvement of SARS-CoV-2 in the COVID-19 course of a patient with prolonged viral RNA detection. This important finding is not representative of the general population since the patient was a 65-year-old woman, and it is well-known that: (1) women are more likely to report frequent symptoms of dry eye; (2) the prevalence of dry eye is relatively higher in Chinese population than that reported for white, and lastly (3) age over 60 has significantly higher prevalence rates (30).

A more recent study cohort conducted by Valente et al. on pediatric patients with confirmed COVID-19 infection hospitalized from 16th Mar to 15th Apr 2020, at the Bambino Gesù Children's Hospital concluded that the ocular manifestations associated with the viral infection appear to have had a milder clinical course in pediatric patients than in adult patients showing the same symptoms (31).

Taken together, both clinical evidence and laboratory test findings suggest that the conjunctiva is rarely involved in SARS-CoV-2 infection since it is naturally protected, so conjunctiva is neither a tissue of choice for SARS-CoV-2 infection and is not a preferential route of entry for the virus to infect the respiratory tract.

These findings are supported by Kumar et al., who performed a study on 45 infected patients. Although the study results showed that SARS-CoV-2 could be detected in conjunctival swabs, the rate of positive detection of SARS-CoV-2 in conjunctival swabs is much lower when compared to nasal ones (32).

A second report on 43 patients with severe COVID-19 published by Karimi et al. (33) on 18th May 2020 demonstrated that ocular manifestation was rare also in patients with severe COVID-19 (7%).

Another study on 36 SARS-CoV-2 patients divided into two groups (18 with conjunctivitis and 18 without) published by Güemes-Villahoz et al. (34) on 24th Jun 2020 concluded that the clinical course was the same regardless of the onset of conjunctivitis.

All these results taken together, suggest that conjunctivitis is not a conditioning factor for SARS-CoV-2 detection in ocular fluids.

## Discussion

### Detection of SARS-CoV-2

In this scenario, although the early detection of infectious SARS-CoV-2 from ocular fluids may represent an important diagnostic advancement useful to counteract the spread of the virus in the community, the mechanisms of virus ocular tropism and how human eye cells could support viral replication is yet to be clarified (35). One of the factors that could explain the very low positive rate of SARS-CoV-2 confirmed by RT-PCR in tears and conjunctival secretions in patients with COVID-19 can be attributed to the low sensitivity of the RT-PCR test currently used for SARS-CoV-2 RNA. The sampling time can also be a determining factor since the virus, and its genetic material may be present in ocular fluids for a short period of the disease. Finally, the low amount of collected tears and conjunctival secretions may also be responsible for PCR negativity (36). On the other hand, the amount of virus or receptor expression necessary to cause infection is not known; however, currently, the eye is not considered to be a high-risk tissue due to the low ACE2 and TMPRSS2 expression (37).

Although ocular manifestations of COVID-19 are currently thought to be self-limited, the necessary condition for the SARS-CoV-2 to be transmitted through the conjunctival epithelial tissue is the ability to replicate in conjunctival cells, inducing cytopathic changes necessary for its identification (38) thus the eye cannot be completely excluded as a transmission route of the infection.

### Healthcare Prevention and Protection

Healthcare personnel and all the people that enter in contact with infected or suspected patient must always use the approved personal protective equipment (PPE). In Italy, a high number of medical doctors and other healthcare professionals have died of COVID-19; this list includes mainly general practitioners but also several dentists and one ophthalmologist. The dramatic situation in which the medical community finds itself mirrors what was reported for the overall deaths related to COVID-19 in Italy. Indeed most of the dead doctors were male. The high rate of infection among nurses and healthcare staff is due to the lack of an adequate number or even the absence of PPE in some hospitals. Besides, very often, nurses and doctors are forced to wear masks inappropriately, thus reducing their effectiveness in containing the spread of the disease.

It is unacceptable to work without sufficient protection, and governments must ensure an adequate and constant supply for all health facilities involved. In this context, already in February 2020, the Italian Ministry of Health had issued the “ASSISTENTIAL ADDRESS LINES OF THE CRITICAL PATIENT AFFECTED BY COVID-19" (39). This document stressed the importance of prescribed PPE such as masks, overalls, long gloves and visors to protect the ocular mucous membranes from the moment the patient is admitted and during all those procedures that could generate aerosols (i.e., endotracheal aspiration, intubation, tracheostomy, bronchoscopy, central venous catheter placement in the jugular, subclavian or femoral vein or during cardiopulmonary resuscitation). Lastly, taking into account the proximity to patients during eye examination make ophthalmologists at risk for droplet transmission; moreover, the unavoidable physical contact they have with patients' eyes, results in increased susceptibility through direct contact (40). For these reasons strict hand hygiene and PPE are highly recommended for health care workers to avoid hospital-related viral transmission during ophthalmic practice.

## Author Contributions

GPP conceptualized and wrote the manuscript. RDM edited the microbiological findings and CC edited the ophthalmological implications.

## Conflict of Interest

The authors declare that the research was conducted in the absence of any commercial or financial relationships that could be construed as a potential conflict of interest.
